# Functional mechanism and clinical implications of *MicroRNA‐423* in human cancers

**DOI:** 10.1002/cam4.3557

**Published:** 2020-11-11

**Authors:** RuiSheng Ke, LiZhi Lv, SiYu Zhang, FuXing Zhang, Yi Jiang

**Affiliations:** ^1^ Department of General Surgery The First Affiliated Hospital of Xiamen University Xiamen China; ^2^ The Fuzong Clinical Medical College of Fujian Medical University Fuzhou City China; ^3^ Department of Hepatobiliary Surgery 900 Hospital of the Joint Logistics Team Fuzhou City China

**Keywords:** clinical implications, functional mechanism, *MicroRNA‐423*, tumorigenesis

## Abstract

MicroRNAs play a vital role in the regulatory mechanisms of tumorigenesis. Current research indicates that *microRNA‐423* (*miR‐423*) is abnormally expressed in various human tumors and participates in multiple signaling pathways of cancer progression. In most studies, *miR‐423* was confirmed as oncomiR, while a few contradictory reports considered *miR‐423* as an anticancer *miRNA*. The paradoxical role in cancer may hinder the application of *miR‐423* as a diagnostic and therapeutic target. Simultaneously, the interaction mechanism between *miR‐423* and *lncRNA* also needs attention. In this review, we have summarized the dual role of aberrant *miR‐423* expression and its mechanisms in tumorigenesis, and the therapeutic potential of *miR‐423* in human tumors.

## Graphical abstract

Has‐microRNA‐423 (miR‐423) is abnormally expressed in various human tumors and plays a role in cancer progression. Abnormal expression of miR‐423 may have dual effects, and actively exploring its mechanism in tumorigenesis may contribute to the treatment of tumors. Increasing evidence suggests that miR‐423 may serve as a biomarker for early diagnosis and prognostic monitoring of human cancers.

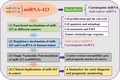

## 1 BACKGROUND

Human cancer is a major public health concern worldwide.^1^ Researchers have accumulated a wealth of knowledge about a complex network of multifunctional regulatory factors that form the basis of cancer progression and the underlying mechanisms, such as sustained proliferation regulation, evading immune surveillance, oncogenes overexpression, inhibition of apoptosis, and the interaction with proteins that play a synergistic effect in carcinogenesis and metastasis.^2^, ^3^

Dysregulation of microRNAs (miRNAs) in human cancers has been significantly involved in various signaling pathways and the pathogenesis.^4^, ^5^, ^6^ These molecules are a type of small non‐coding RNA, which regulate the expression of certain protein‐coding genes.^7^, ^8^ MiRNAs function as transcriptional regulators by forming RNA‐induced silencing complexes or as competitive endogenous RNAs (ceRNA).^9^, ^10^ That specific regulatory mechanisms of miRNAs may affect tumorigenesis and may be potential targets for gene therapy.^11^ MiRNAs usually act post‐transcriptionally by base pairing with the 3'untranslated region of the mRNA to regulate protein synthesis.^12^ The binding sites of miRNA targeting mechanism mRNAs are enriched, which endows miRNA itself with strong and specific feedback regulation ability to miRNA targeting activity, that is, the fragment‐specific feedback of miRNA pathway is automatically regulated, complementary with transcription factors to form a mixed feedback loop.^13^ Exosomes are membrane‐derived vesicles (30–100 nm) secreted in body fluids, which can selectively transmit miRNA through intercellular communication. *exosomal‐derived miRNAs* are transferred to receptor cells, and they regulate the expression of target genes,^14^ and they also can participate in the development of chemical resistance.^15^ There is much evidence that dysregulated expression of various miRNAs in cells can ultimately enhance chemoresistance by affecting cell survival, apoptosis, and autophagy, thereby pushing normal cells toward cancerous cells.^16^ In some cases, aberrant expression of miRNAs has been shown to be associated with tumor metastasis. These miRNAs regulate metastasis by regulating the formation of metastatic niches and by regulating their target genes for metabolic reprogramming.^17^ As regulators of the human genome,^18^ the vast majority of miRNAs are located in cancer‐related genomic regions and participate in tumor progression in the form of tumor suppressor genes or proto‐oncogenes.^19^ In addition, some miRNAs have been identified as clinical biomarkers of certain human cancers.^20^, ^21^ Further elucidation of the associations between miRNAs and carcinogenesis as well as clarification of the underlying mechanisms may contribute to an in‐depth understanding of the mechanisms of cancer development and highlight therapeutic targets for use in the clinic.^22^

*MicroRNA‐423* (*miR‐423*) is located at 17q11.2 of chromosome 17 (gene coding ID: 494335). Two mature sequences (namely *miR‐423‐3p* and *miR‐423‐5p*) of the *miRNA‐423* gene were identified by searching the miRBase sequences.^23^, ^24^, ^25^ Previous studies have confirmed the differential expression of *miR‐423* in various human tumors,^26^ and also indicated differences in the functional mechanisms in different tumor types.^27^, ^28^ In this article, we briefly review the latest reports on the functional mechanisms of *miR‐423* in tumors and its involvement in signaling pathways. The potential value of single nucleotide polymorphisms (*SNPs*) in *pre‐miR‐423* (*rs6505162*) as a diagnostic biomarker, and a therapeutic target, are also discussed.

## 2 THE FUNCTIONAL MECHANISMS OF *MiR‐423* IN DIFFERENT CANCERS

MiRNA dysregulation is caused by genetic factors and epigenetic changes.^29^ MiRNAs are engaged in fundamental cellular processes that involve evolutionarily conserved cascade reactions.^22^ *MiR‐423* can function as an suppressor or oncogene gene in cancer via a large number of direct and indirect regulatory target genes (Table 1). Numerous studies have indicated that *miR‐423* has different expression patterns and distinct functions in cellular processes such as cell proliferation and cell cycle, cell autophagy and apoptosis, tumor metastasis, and chemoresistance. Therefore, *miR‐423* is implicated as a good target for further investigations aimed at designing novel therapeutic strategies to manage tumors.

**TABLE 1:** Disease/conditions in which miR‐423 are involved and the possible targets of miR‐423 in human tumors

Tumors type	miR‐423 form	miR‐423 expression alteration and functional effects	Function or validated targets	Biological functions of	References
Gastric cancer (GC)	*miR‐423‐5p*	Up, carcinogenic	*SUFU(−)*	Cell proliferation and metastasis(+)	31
	*miR‐423‐5p*	Up, carcinogenic	*TFF1(−)*	Cell proliferation and invasion(+)	34
	*miR‐423‐3p*	Up, carcinogenic	*Bim(−), Beclin‐1(+) and Atg 7(−)*	Cell proliferation, migration and invasion(+), autophagy(−)	52
	*miR‐423‐5p*	Down, anti‐cancer	*β‐catenin Interference(+)*	Cell proliferation(−), apoptosis(+), sensitivity to 5‐fluorouracil(+)	50
Hepatocellular carcinoma (HCC)	*miR‐423*	Up, carcinogenic	*p21Cip1/Waf1(−)*	Cell growth and regulates G1/S transition(+)	25
	*miR‐423‐5*	Up, carcinogenic	*ATG7(+), pErk‐1/2 and AKT(−)*	Cell proliferation(+), autophagy (+), chemoresistance(+)	44
	*miR‐423*	Up, carcinogenic	*BRMS1(−)*	Cell invasiveness(+)	56
Glioblastomas (GBM)	*miR‐423*	Up, carcinogenic	*ING‐4(−), p‐AKT and p‐ERK1/2(+)*	Cell proliferation, angiogenesis, invasion, chemoresistance(+)	37
	*miR‐423‐3p*	Up, carcinogenic	*PANX2(−)*	Cell proliferation and apoptosis(+)	46
	*miR‐423‐5p*	Up, carcinogenic	*mitochondrial pathway(+), Bax/Bcl‐2 and cytochrome c(+), Apaf‐1 and caspase‐3(+)*	Cell apoptosis(−), cell growth(+),sensitivity to apigenin(+)	47
	*miR‐423‐5p*	Down, anti‐cancer	*PLCB1(−), p‐ERK(+)*	Cell Proliferation and metastasis(−), apoptosis(+)	48
Laryngeal carcinoma (LYC)	*miR‐423‐3p*	Up, carcinogenic	*AdipoR2(−)*	Cell proliferation, clonogenicity, migration and invasion.(+)	57
Lung adenocarcinoma (LAD)	*miR‐423‐5p*	Up, carcinogenic	*MTSS1(−)*	Colony formation, cell motility, migration and invasion(+)	60
Lung cancer (LC)	*miR‐423‐3p*	Up, carcinogenic	*TDP‐43 regulates(+)*	Cell proliferation, migration and invasion(+)	58, 59
Colorectal cancer (CRC)	*miR‐423‐5p*	Down, anti‐cancer	*caspases 3, 8 and 9, p53 (+); caspase‐dependent*	Cell apoptosis(+), cancer growth(−)	49
	*miR‐423‐5p*	Down, anti‐cancer	*LAMC1(+)*	Cell migration and invasion(−), genetic modifier	62, 63
	*miR‐423‐3p*	Up, carcinogenic	*p21Cip1/Waf1(−)*	Cell cycle and cell proliferation(+)	43
Breast cancer (BC)	*miR‐423*	Up, carcinogenic	*PCNA and mutant p53(+)*	Cell proliferation(+)	36
	*miR‐423*	Up, carcinogenic	*ZFP36(−), Wnt /β‐catenin(+)*	Chemoresistance(+), migration and proliferation(+), apoptosis(−)	68
	*miR‐423*	Up, carcinogenic	*TNIP2(−), NF‐κB signaling pathway(+)*	Invasiveness(+)	64
Ovarian cancer (OC)	*miR‐423‐5p*	Down, anti‐cancer	‐	Cell proliferation, colony formation and invasion(−)	38
Endometrial cancer (EC)	*miR‐423*	Up, carcinogenic	*caspase 3/7 and Bcl‐2(−), PTEN(−) and p‐AKT(+)*	Cell proliferation, migration and invasion(+), EMT(+), chemoresistance(+), apoptosis(−)	45, 55
Prostate cancer (PC)	*miR‐423‐5p*	Up, carcinogenic	*GRIM‐19(−), BCL‐2(+)*	Proliferation(+), apoptosis(−)	35
Osteosarcoma	*miR‐423‐5p*	Down, anti‐cancer	*STMN1(−)*	Cell proliferation, colony formation and invasion(+)	40
Cervical cancer (CC)	*miR‐423‐3p*	Down, anti‐cancer	*Endogenous E6/E7‐regulated(+)*	Cell proliferation(+), senescence and apoptosis(−)	39

Abbreviations: (−), suppression and reduction; (+), promotion and activation; AdipoR2, adiponectin receptor 2; Atg7, autophagy‐related gene 7; Bim, Bcl‐2‐interacting mediator; BRMS1, breast cancer metastasis suppressor 1; EMT, epithelial‐mesenchymal transition; FOSL2, FOS like 2, AP‐1 transcription factor subunit; ING‐4, inhibitor of growth 4; LAMC1, laminin γ1; MTSS1, metastasis suppressor 1; p53, tumor protein 53; PANX2, pannexin 2; PCNA, proliferating cell nuclear antigen; PLCB1, phospholipase C beta 1; PTEN, phosphatase and tensin homolog; SUFU, suppressor of fused homolog; TDP‐43, TAR DNA binding protein 43; TFF1, trefoil factor 1.

### 2.1 Mechanisms of *miR‐423* in tumor cell proliferation and the cell cycle

**FIGURE 1:**
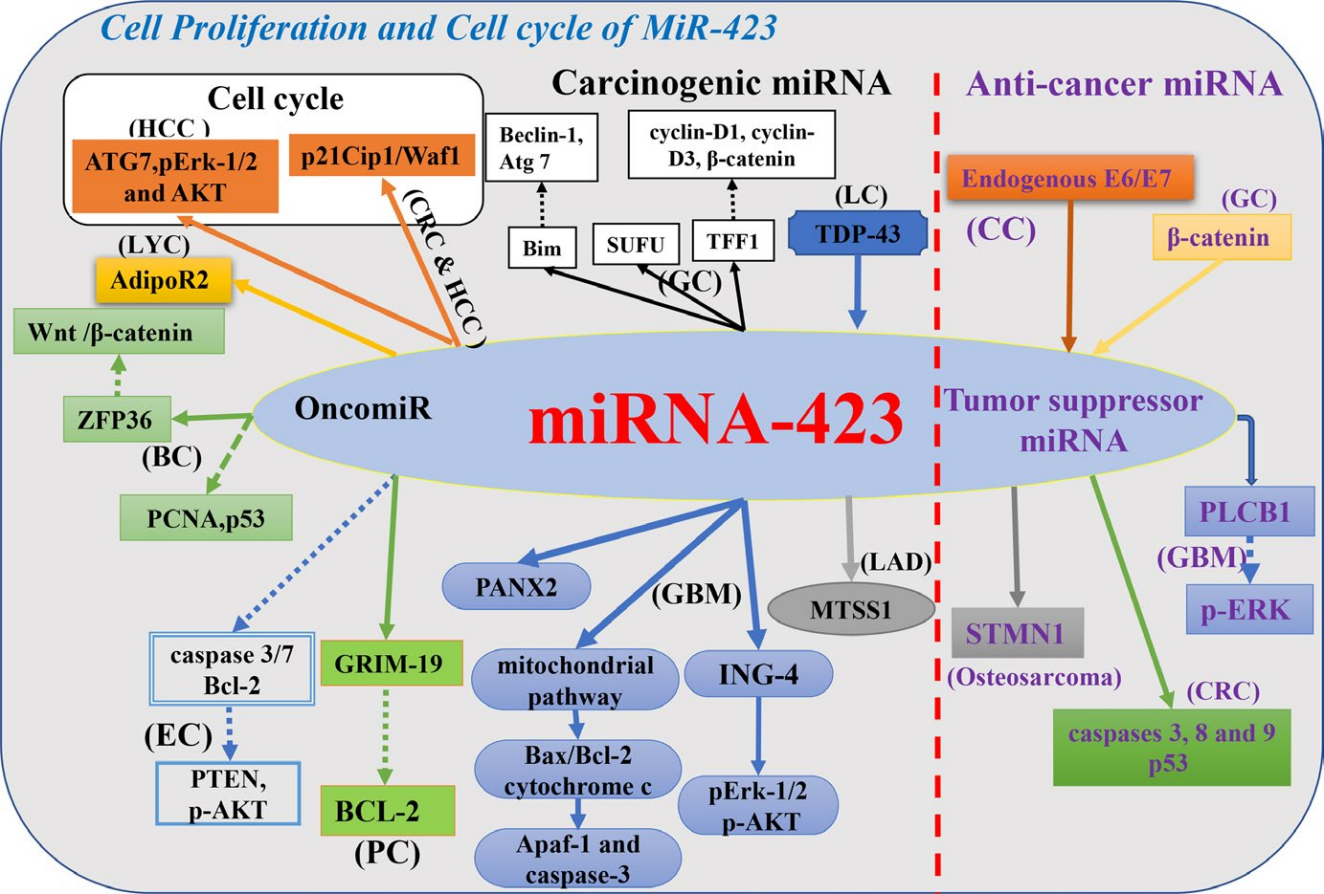
Schematic view of different roles of *miR‐423* in cancer cell proliferation and experiments have confirmed that *miR‐423* is involved in the regulation of cellular targets and pathways in tumor cell proliferation and cell cycle. Black font indicates that *miR‐423* acts as a carcinogenic miRNA effect in tumors; purple font indicates that *miR‐423* acts as a tumor suppressor miRNA effect in tumors. (Tumor name abbreviation: BC, breast cancer; CC, cervical cancer; CRC, colorectal cancer; EC, endometrial cancer; GBM, glioblastoma; GC, gastric cancer; HCC, hepatocellular carcinoma; LAD, lung adenocarcinoma; LC, lung cancer; LYC, laryngeal carcinoma; NPC, nasopharyngeal carcinoma; OC, ovarian cancer; PC, prostate cancer; PTC, papillary thyroid carcinoma.)

The ability to maintain unrestricted cell proliferation is a hallmark of human tumors.^22^, ^30^ The uptake of exosomes rich in *miR‐423‐5p* by cancer cells leads to gastric cancer progression by directly targeting the *suppressor of fused protein(SUFU)*.^31^, ^32^ Deletion of *Trefoil factor 1 (TFF1)* protein promotes *β‐catenin* activation and gastric tumorigenesis,^33^ while *miR‐423‐5p* expression targets negative regulation *TFF1* expression and participated in proliferation and metastasis‐related processes of gastric cancer cells.^34^ Furthermore, inhibition of *miR‐423‐5p* expression upregulates gene associated with *GRIM‐19* expression and decreases *BCL‐2* expression to inhibit prostate cancer cell proliferation and promote apoptosis.^35^ *Pre‐miR‐423‐12C* treatment inhibited breast cancer (BC) cell proliferation to a greater extent than *pre‐miR‐423‐12A*, resulting in the proliferative capacity in the *pre‐miR‐423‐12C* BC group was worse than that in the *pre‐miR‐423‐12A* group.^36^ Furthermore, the *miR‐423* chain promotes BC cell growth by enhancing the expression of mutant *p53* and *proliferating cell nuclear antigen (PCNA)*.^36^ Overexpression of *miR‐423‐5p* induces upregulation of *p‐ERK1/2* and *p‐AKT* and enhances glioma cell proliferation, as well as angiogenesis and metastasis by targeting *inhibitor of growth 4 (ING‐4)*.^37^ *MiR‐423* has also been found to possess a tumor suppressor gene in a few studies, which can inhibit cell proliferation of tumors. Ectopic expression of *miR‐423‐5p*, as an anticancer *miRNA*, inhibits ovarian cancer cell proliferation and colony formation.^38^ Downregulation of *miR‐423‐3p* in exosomes secreted by HeLa cervical cancer cells results in *E6/E7*‐dependent cell proliferation in HPV‐positive cancer cells.^39^ *MiR‐423‐5p* mediated cell colony formation and proliferation in osteosarcoma by inhibiting *stathmin1 (STMN1)* expression.^40^ Thus, *miR‐423* is implicated as a therapeutic target to inhibit the proliferation of tumor cells and prevent tumor progression (Figure 1).

The normal cell division process involves a series of highly regulated cell cycle steps.^30^ Blockade of this process at certain stages can cause cancer.^22^ Bioinformatics analysis has pointed out that *miR‐423‐5p* mainly acted as oncomiR in tumors and involved in the cancer cell cycle.^41^ *MiR‐423‐3p* can also affect cell cycle progression by regulating ATP/energy metabolism.^42^ *MiR‐423‐3p* promotes the G1 phase to S phase cell cycle transition and inhibits the colorectal cancer cells proliferation by inhibiting *p21Cip1/Waf1*.^43^ *MiR‐423‐3p* has similar functional effects in hepatocellular carcinoma (HCC), whereas *miR‐423‐5p* does not.^25^ Interestingly, after *miR‐423‐5p* was up‐regulated in HCC cells, it was found that the percentage of HCC cells in the S phase showed an increase.^44^ The ability of *miR‐423* to regulate the cell cycle in tumors remains to be clarified (Figure 1).

### 2.2 Mechanisms of *miR‐423* in apoptosis and autophagy

**FIGURE 2:**
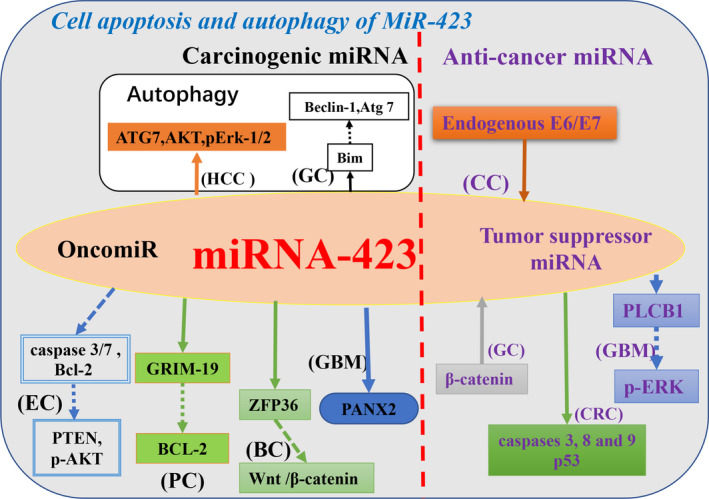
Schematic view of different cancer roles of *miR‐423* in cell apoptosis and autophagy, and experimentally confirmed cellular targets and pathways of *miR‐423* in the cell apoptosis and autophagy. Black font indicates that *miR‐423* acts as a carcinogenic miRNA effect in tumors; purple font indicates that *miR‐423* acts as a tumor suppressor miRNA effect in tumors. (Tumor name abbreviation: BC, breast cancer; CC, cervical cancer; CRC, colorectal cancer; GBM, glioblastoma; GC, gastric cancer; HCC, hepatocellular carcinoma; LAD, lung adenocarcinoma; PC, prostate cancer.)

Inhibition of apoptosis can promote tumor progression. Conversely, apoptosis induction can inhibit tumor progression.^22^ A study showed that *miR‐423* inhibits cisplatin‐induced apoptosis by modulating the expression levels of *caspase 3/7 and Bcl‐2* in endometrial cancer cells.^45^ *Pannexin 2 (PANX2)*, which is considered to be a target gene of *miR‐423‐3p*, is upregulated in glioma cells when *miR‐423‐3p* is inhibited, leading to a reduction in glioma cell proliferation and the induction of apoptosis.^46^ Similarly, *miR‐423‐5p* targets inhibition of *ING‐4* to inhibit apoptosis of glioblastoma cells.^37^ Apigenin is a plant‐derived flavonoid that participates in numerous signaling cascades in tumors.^3^ When combined with the inhibitory effect of *miR‐423‐5p*, apigenin‐induced glioma stem cell apoptosis was significantly elevated.^3^, ^47^ In‐depth studies have shown that *miR‐423‐5p* enhances the induction of apoptosis by apigenin via the mitochondrial pathway and changes the expression of *Bax/Bcl‐2* and *cytochrome c/Apaf‐1/caspase‐3*.^47^ Inhibition of apoptosis by *miR‐423* in the above studies promoted tumor progression. On the contrary, *miR‐423* can also be used as anti‐cancer miRNA to induce cancer cell apoptosis. For example, *MiR‐423‐5p* inhibits glioma metastasis by targeting *phospholipase C beta 1 (PLCB1)* via the *ERK*‐dependent pathway, and also promotes apoptosis in glioma cells.^48^ Increased *miR‐423‐5p* expression induces the expression of caspase 3, 8, 9, and *p53* in colon cancer cells, thereby inhibiting cell proliferation and promoting cell apoptosis.^49^ Similarly, Knockdown expression of *β‐catenin* can downregulate *miR‐423‐5p* expression, thereby promoting gastric cancer apoptosis.^50^ Thus, these reports provide evidence that *miR‐423* influences the process of apoptosis in tumor cells (Figure 2).

Interventions to both stimulate and inhibit autophagy have been proposed as cancer therapies.^51^ So far, there have been few reports on *miR‐423* and autophagy in cancer. Treatment of liver cancer cells with sorafenib showed that *miR‐423‐5p* is a promoter of autophagy in HCC cells.^44^ *MiR‐423‐3p* activates carcinogenesis of gastric cancer cells and affects *Beclin‐1*‐dependent autophagy by downregulating *Bim* and *Atg 7* expression.^52^ It can be speculated that more than one autophagy pathway is influenced by *miR‐423* in different tumors and therefore, further research is needed to elucidate the mechanism of *miR‐423* and cell autophagy in specific tumors (Figure 2).

### 2.3 *MiR‐423* and metastasis

**FIGURE 3:**
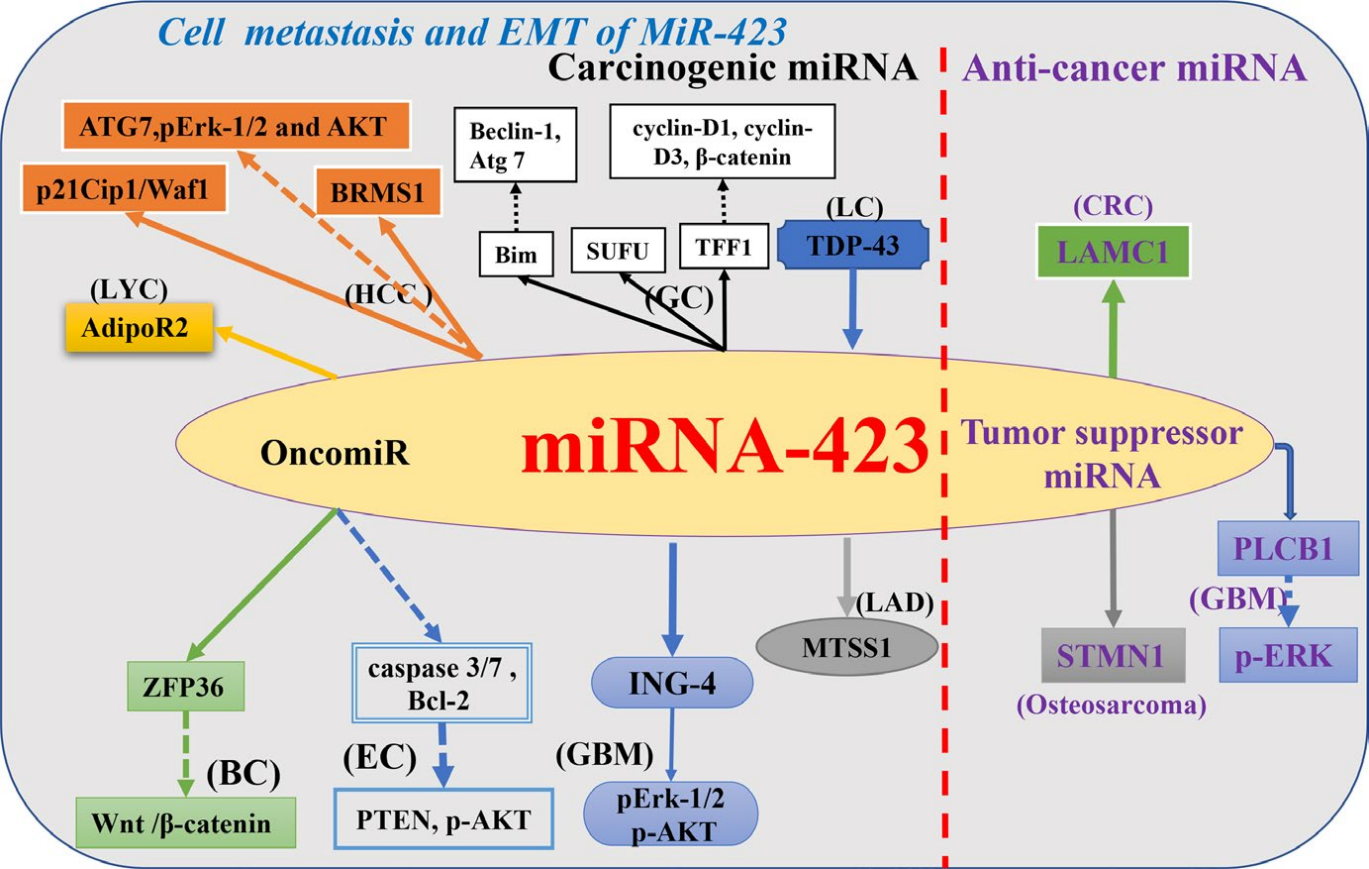
Schematic view of different cancer roles of *miR‐423* in cancer metastasis (including EMT, migration and invasion) and experimentally confirmed cellular targets and pathways of *miR‐423*. Black font indicates that *miR‐423* acts as a carcinogenic miRNA effect in tumors; purple font indicates that *miR‐423* acts as a tumor suppressor miRNA effect in tumors. (Tumor name abbreviation: BC, breast cancer; CC, cervical cancer; CRC, colorectal cancer; EC, endometrial cancer; GBM, glioblastoma; GC, gastric cancer; HCC, hepatocellular carcinoma; LAD, lung adenocarcinoma; LC, lung cancer; LYC, laryngeal carcinoma; NPC, nasopharyngeal carcinoma; OC, ovarian cancer; PC, prostate cancer; PTC, papillary thyroid carcinoma.)

*MiR‐423* is a common marker of transcarcinal metastasis in metastatic samples.^53^ Epithelial‐mesenchymal transition (EMT) facilitates the smooth colonization of cancer cells in other target organs.^54^ Overexpression of *miR‐423* in endometrial cancer cells alters EMT and modulates the expression levels of *PTEN* and *p‐AKT*, ultimately promoting the metastasis of endometrial cancer cells.^45^, ^55^ *MiRNA‐423‐5p* participates in gastric cancer (GC) cell invasion by downregulating *TFF1* expression.^34^ Similarly, the *miR‐423‐3p‐Bim* axis promotes GC cell metastasis.^52^ *MiR‐423* also significantly inhibited *BRMS1* protein expression and exacerbated the aggressiveness of HCC.^56^ In laryngeal cancer, *miR‐423‐3p* has been confirmed to affect tumor cell metastasis by regulating *AdipoR2*.^57^ Furthermore, *TAR DNA binding protein (TDP‐43)* promotes lung cancer cell metastasis by upregulating the expression of oncogenic *miR‐423‐3p*.^58^, ^59^ In addition to these functions, *miR‐423‐5p* also significantly promotes colony formation, cell migration, invasion and distant brain metastasis in lung adenocarcinoma by inhibiting *metastasis suppressor 1 (MTSS1)* expression,^60^ Similarly, high *miR‐423‐3p* expression is also associated with an increased risk of brain metastases from lung adenocarcinoma.^61^ In addition, overexpression of *miR‐423‐5p* downregulates *ING‐4* expression in glioma tissues to enhance angiogenesis and promote glioma cell invasion.^37^ With this just the opposite is, *MiR‐423* can also act as a tumor suppressor in colon cancer.The up‐regulated *MiR‐423‐5p* can specifically bind and downregulate the expression of *LAMC1*, thereby inhibiting the migration of colorectal cancer cells.^62^, ^63^ For another example, *miR‐423‐5p* overexpression inhibits osteosarcoma cell metastasis by inhibiting *STMN1* expression.^40^ Similarly, *miR‐423‐5p* directly targets *PLCB1* expression in glioma to inhibit tumor cell metastasis.^48^ The latest research shows that abnormally high expression of *miR‐423* enhances the aggressiveness of breast cancer cells. Mechanistically, the up‐regulation of *miR‐423* targets the inhibition of *TNIP2* expression resulting in the activation of the *NF‐κB* signaling pathway and the increased expression of *snails* and *twists*.^64^ Thus, it is clear that abnormal expression of *miR‐423* can affect cancer cell metastasis of human tumors, miR‐423 may be a promising prognostic and therapeutic marker for patients with metastatic cancer. (Figure 3).

### 2.4 *MiR‐423* and chemotherapy response

**FIGURE 4:**
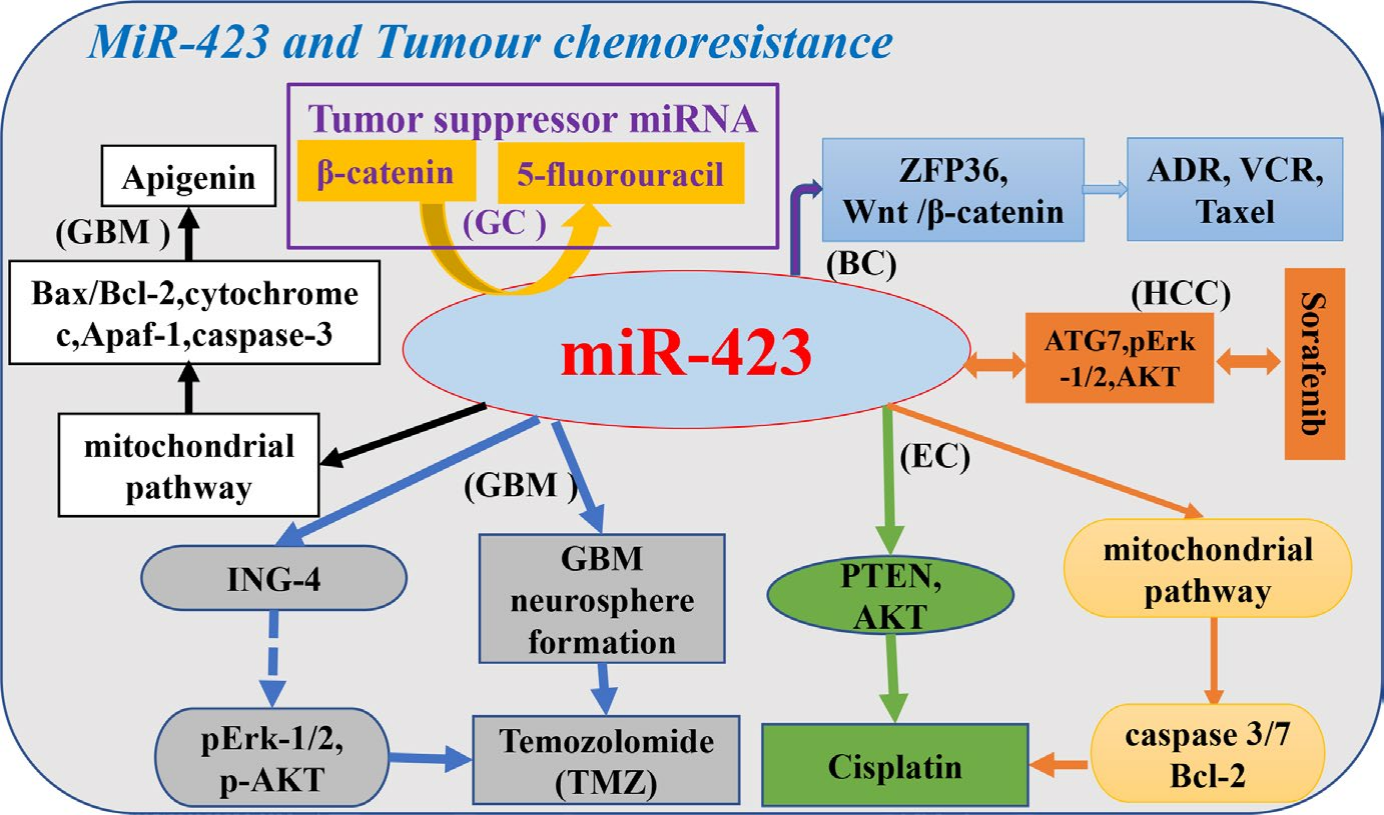
Schematic view of different cancer roles of *miR‐423* in chemotherapy response and experimentally confirmed cellular targets and pathways of *miR‐423* in chemotherapy response. Black font indicates that *miR‐423* acts as a carcinogenic miRNA effect in tumors; purple font indicates that *miR‐423* acts as a tumor suppressor miRNA effect in tumors. (Tumor name abbreviation: EC, endometrial cancer; GBM, glioblastoma; GC, gastric cancer; HCC, hepatocellular carcinoma.)

Resistance to chemotherapeutic drugs impairs the ability to cure cancer patients after relapse.^22^ In malignant tumors, *Wnt/β‐catenin* signaling pathway can regulate certain miRNAs to participate in malignant tumor progression.^50^, ^65^ Decreasing *β‐catenin* expression downregulates *miR‐423‐5p* expression and reduces the resistance of gastric cancer cells to 5‐fluorouracil.^50^ *miR‐423‐5p* may act as a tumor suppressor in gastric cancer, and upregulating its expression can increase the sensitivity of gastric cancer cells to 5‐fluorouracil. Whole blood samples stored at room temperature for 24 hours had no significant effect on the concentration of *miR‐423‐5p*, indicating that serum *miR‐423‐5p* can be used as a biomarker for therapeutic response.^66^ In sorafenib‐treated HCC, the upregulated levels of secreted *miR‐423‐5p* correlated positively with the sorafenib response.^44^ Concurrently, *miR‐423‐5p* transfected HCC cells can promote the regulation of autophagy.^44^ Ectopic *miR‐423‐5p* highly expression in gliomas is a potential tumor promoter, targets *ING‐4* expression, enhances the formation of glioblastoma neurospheres and confers resistance to temozolomide in glioma cells.^37^, ^67^ Furthermore, knockdown *miR‐423‐5p* increases the sensitivity to apigenin‐induced apoptosis in glioma stem cells,^47^ the combination of apigenin and *miR‐423‐5p* knockdown has an additive effect of promoting apoptosis and inhibiting cell proliferation.^47^ *MiR‐423* high expression induces the sensitivity of endometrial cancer cells to cisplatin by causing loss of *PTEN* and activation of *AKT*. Therefore, *miR‐423* is implicated as a predictive biomarker of cisplatin chemotherapy response in endometrial cancer.^45^ *MiR‐423* has been proved to be an oncogene that promotes the malignant breast cancer (BC) cells proliferation and migration, can inhibit the expression of *ZFP36* in BC cells, and activate *Wnt/β‐catenin* signaling pathway, ultimately affecting the chemoresistance and apoptosis rate of BC cells.^68^ Similarly, the abnormal exosomal *miR‐423‐5p* expression affects the therapeutic effect of cisplatin in triple negative BC.^69^ Meanwhile, circulating miR‐423‐5p may also serve as a potential biomarker for chemotherapy‐related cardiac dysfunction in BC patients.^70^ There are few reports on the relationship between *miR‐423* and chemotherapy response in tumors. Thus, the association of *miR‐423* with chemotherapy resistance in other tumors remains to be clarified (Figure 4).

## 3 THE REGULATORY MECHANISM OF *MiR‐423* AND LNCRNA IN HUMAN TUMOR

Long noncoding RNA (lncRNAs) is a new regulator of genomic stability and human diseases.^71^ lncRNAs plays an essential role in maintaining embryonic stem cells pluripotency, coordinating the regulation of immune cell activation and heterochromatin formation.^72^ Many factors have been found to affect the expression and secondary structure of lncRNAs.^73^ The different processing and localization of lncRNA are the factors that lead to the rapid evolution of lncRNA's functions, which contribute to the nonconservative functions of stem cells.^74^ Unstable transcripts associated with lncRNAs and promoters or enhancers are preferentially localized to chromatin, some of which regulate chromatin structure, transcription, and RNA processing.^75^ The data show that lncRNA potency can be closely related to lncRNA abundance. In the lncRNA targeted domain, Polycomb responsive complexes are recruited to CpG island through lncRNA independent mechanism, and then interact with lncRNA and its related proteins through three‐dimensional space to make Polycomb responsive complexes diffuse and nucleate in lncRNA targeted domain.^76^ Many lncRNAs related to various types of cancer have been identified by genome‐wide association studies of tumor samples. LncRNA expression mutations can promote tumor occurrence and metastasis.^77^ LncRNA also participates in the metabolic remodeling of tumor cells and promotes the rapid proliferation of cancer cells.^72^ Numerous studies have shown that lncRNAs can bind to miRNA sites as competitive endogenous RNAs (ceRNAs), thereby regulating and affecting the expression of downstream target genes. These lncRNAs can play an important regulatory role in tumor pathogenesis through the lncRNA‐microRNA‐mRNA related ceRNA network.^78^

**FIGURE 5:**
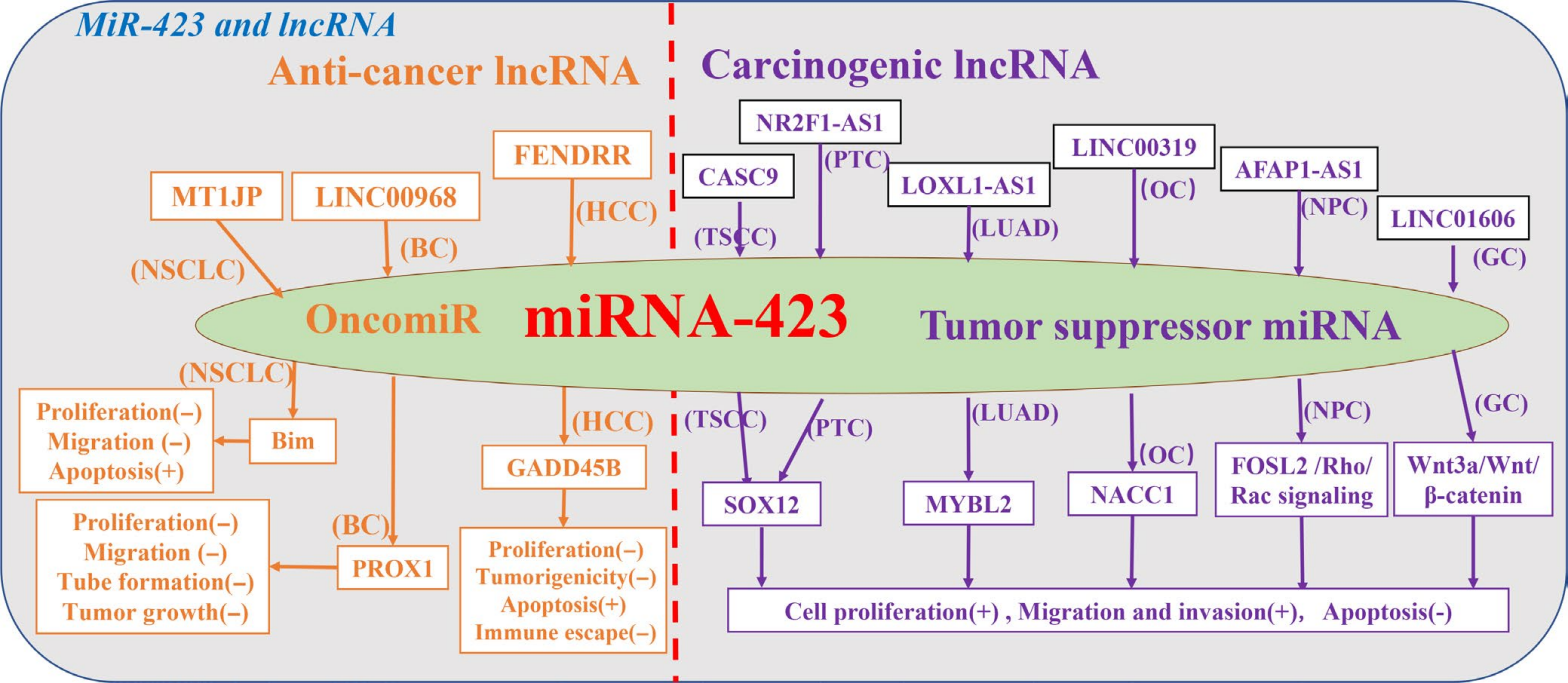
Regulatory mechanism of *miR‐423* and LncRNA in human tumor. The regulatory roles of *miR‐423* and LncRNAs in tumors are mainly ceRNAs mechanism, which participate in cancer cell proliferation and growth, migration and invasion, apoptosis, immune escape and chemotherapeutic resistance by regulating multiple targets. Orange font indicates that lncRNA plays an Anti‐cancer lncRNA effect in tumors and that *miR‐423* acts as an oncogenic miRNA; purple font indicates that lncRNA acts as a carcinogenic lncRNA effect in tumors and that *miR‐423* acts as a tumor suppressor miRNA. (Tumor name abbreviation: BC, breast cancer; ccRCC, clear cell renal cell carcinoma; GC, gastric cancer; HCC, hepatocellular carcinoma; LAD, lung adenocarcinoma; LC, lung cancer; LUAD, Lung adenocarcinoma; NPC, nasopharyngeal carcinoma; NSCLC, Non‐small cell lung cancer; OC, ovarian cancer; PTC, papillary thyroid carcinoma; TSCC, Tongue squamous cell carcinoma.)

The regulatory role between *miR‐423* and LncRNA in tumors is mainly the ceRNAs mechanism, and we also give a brief overview of the related research progress (Table 2). In some studies, low expression of lncRNA in cancer tissues has been found to act as a tumor suppressor gene and inhibit tumor progression. *LncRNA MT1JP* inhibits the malignant phenotype of non‐small cell lung cancer by regulating the *miRNA‐423‐3p/Bim* signaling axis.^79^ The *lncRNA FENDRR* inhibits *miR‐423‐5p* and upregulates *GADD45B* to reduce proliferation and tumorigenicity and promote apoptosis of hepatoma cells, thereby preventing Treg‐mediated immune escape of liver cancer.^80^ *LncRNA LINC00968* inhibits *miR‐423‐5p* expression in a concentration‐dependent manner and mediates upregulation of *prospero homeobox 1 (PROX1)* expression to inhibit breast cancer progression, such as cell proliferation, migration, and tube‐forming ability as well as tumor growth.^81^ *miR‐423* is highly expressed in the above studies and plays a role of carcinogenic (Figure 5).

**TABLE 2:** Summary of studies on regulatory mechanisms between miR‐423 and lncRNA in human tumors

Tissue or cell type	LncRNA name (*ceRNA yes or no*)	LncRNA alteration and functional effects	LncRNA biological functions	MiR‐423 form and signal axis	miR‐423 functional effects and expression alteration	References
Non‐small cell lung cancer (NSCLC)	*MT1JP (ceRNA)*	Up, anti‐cancer	Cell proliferation(−), cell migration and invasion(−), cell apoptosis(+)	*miRNA‐423‐3p/Bim*	Carcinogenic, down	79
Hepatocellular carcinoma (HCC)	*FENDRR (ceRNA)*	Down, anti‐cancer	Cell proliferation and tumorigenicity(−), apoptosis(+), Treg‐Mediated Immune Escape(−)	*miR‐423‐5p/GADD45B*	Carcinogenic, up	80
Breast cancer (BC)	*LINC00968 (ceRNA)*	Down, anti‐cancer	Cell proliferation, migration and tube formation abilities, tumor growth(−)	*miR‐423‐5p/PROX1*	Carcinogenic, up	81
Tongue squamous cell carcinoma (TSCC)	*CASC9 (ceRNA)*	Up, carcinogenic	Cell proliferation, migration and invasion(+)	*miR‐423‐5p/SOX12*	Anti‐cancer, down	82
Lung adenocarcinoma (LUAD)	*LOXL1‐AS1 (ceRNA)*	Up, carcinogenic	Cell proliferation(+), cell migration and invasion(+), cell apoptosis(−)	*miR‐423‐5p/MYBL2*	Anti‐cancer, down	83
Papillary thyroid carcinoma (PTC)	*NR2F1‐AS1 (ceRNA)*	Up, carcinogenic	Cell proliferation(+), cell migration and invasion(+)	*miR‐423‐5p/SOX12*	Anti‐cancer, down	84
Ovarian cancer (OC)	*LINC00319 (ceRNA)*	Up, carcinogenic	Cell proliferation(+), cell migration and invasion(+)	*miR‐423‐5p/NACC1 pathway*	Anti‐cancer, down	85
Nasopharyngeal carcinoma (NPC)	*AFAP1‐AS1 (ceRNA)*	Up, carcinogenic	Cell migration and invasion(+)	*miR‐423‐5p, FOSL2/Rho/Rac signaling*	Anti‐cancer, down	86
Gastric cancer (GC)	*LINC01606 (ceRNA)*	Up, carcinogenic	Cell migration and invasion(+)	*miR‐423‐5p, Wnt3a/Wnt/β‐catenin signaling*	Anti‐cancer, down	87
Clear cell renal cell carcinoma (ccRCC)	*LINC01426*	Up, carcinogenic	Cell proliferation and migration(+)	*IGF2BP1/CTBP1/HDAC2/miR‐423‐5p/FOXM1 axis*	Anti‐cancer, down	88

Abbreviations: (−), suppression and reduction; (+), promotion and activation; Bim, BCL2‐like 11; FOSL2, FOS like 2, AP‐1 transcription factor subunit; FOXM1, forkhead box M1; GADD45B, growth arrest and DNA damage inducible beta; IGF2BP1, insulin‐like growth factor 2 mRNA‐binding protein 1; MYBL2, myb proto‐oncogene like 2; NACC1, nucleus accumbens associated 1; PLCB1: phospholipase C beta 1; PROX1, prospero homeobox 1; SOX12, SRY (sex determining region Y)‐box 12; Wnt3a, Wnt family member 3A.

With this just the opposite is, *LncRNAs* interacting with *miR‐423* are highly expressed in most tumor tissues and can promote tumor progression. *LncRNA CASC9* is upregulated in tongue squamous cell carcinoma tissues and cells, suggesting that patients have poor prognosis and can influence the malignant process of tongue squamous cell carcinoma by regulating the *miR‐423‐5p/SOX12* axis.^82^ The positive feedback loop of *lncRNA LOXL1 antisense RNA 1(LOXL1‐AS1)/miR‐423‐5p/MYBL2* in lung adenocarcinoma is carcinogenic, and The high expression of *LOXL1‐AS1* competitively inhibits *miR‐423‐5p* expression and targets *MYBL2* to promote proliferation and migration, and inhibit apoptosis of lung adenocarcinoma cell.^83^ The highly expressed *lncRNA NR2F1‐AS1* regulates *miR‐423‐5p/SRY‐box 12* axis to promote papillary thyroid carcinoma cell proliferation and invasion.^84^ Upregulation of *LINC00319* has carcinogenic effects and enhances the expression of *nucleus accumbens associated 1 (NACC1)* by inhibiting *miR‐423‐5p*, which then promotes ovarian cancer cells metastasis and proliferation.^85^ The *lncRNA AFAP1‐AS1* competitively inhibits *miR‐423‐5p* expression in nasopharyngeal carcinoma cells to regulate the *Rho/Rac* pathway, thereby mediating the expression of *FOSL2* and ultimately, promoting the distant metastasis of nasopharyngeal carcinoma cells.^86^ The high expression of *LINC01606* in gastric cancer (GC) liberates *miR‐423‐5p* bound to *Wnt3a* through a *competitive endogenous RNA (ceRNA)* mechanism, thereby upregulating Wnt3a expression and inhibiting the *Wnt/β‐catenin* pathway to promote gastric cancer cell metastasis.^87^ *LINC01426* is upregulated in the renal clear cell carcinoma (ccRCC) cell line, promoting the proliferation and migration of rccRCC cells.^88^ Mechanistically, *LINC01426* upregulates *FOXM1* expression by inhibiting *miR‐423‐5p*, while *LINC01426* can recruit *IGF2BP1* to increase *CTBP1* expression. *CTBP1* and *HDAC2* can also inhibit *miR‐423‐5p* expression, while downregulated *miR‐423‐5p* can Targeted upregulation of *FOXM1* expression to promote the progress of ccRCC.^88^ In the above studies, it was found that the expression of *miR‐423* was often inhibited and played an anti‐tumor role (Figure 5).

In summary, compared with *miR‐423‐3p*, the functions and molecular mechanisms of *miR‐423‐5p* in human tumors have been more extensively investigated and may be more important, although this speculation remains to be confirmed in studies. *MiR‐423* interacts with other biomolecules and participates in the tumor progression. Thus, *miR‐423* is implicated as a biological target for tumor therapy.

## 4 PROGRESS IN SINGLE NUCLEOTIDE POLYMORPHISM (SNP) RESEARCH ON *MiR‐423* (rs6505162)

Due to the inherent cancer‐specific heterogeneity, SNPs play a crucial role in the development of human tumors.^89^, ^90^ A meta‐analysis of *miR‐423 polymorphisms* and cancer prognosis suggests that *rs6505162* is a prognostic marker of all common human cancers.^91^ According to another meta‐analysis, *rs6505162* may participate in the prevention of cancers such as lung cancer and colorectal cancer, but does not reduce the risk of breast cancer, esophageal cancer and gastric cancer.^92^ These polymorphisms have the potential to lead to metabolic abnormalities and changes in cancer susceptibility (Table 3). The *rs6505162 C>A polymorphism*, mapping to chromosome 17 at 17q11.2, is situated in the *pre‐miRNA* region of *miR‐423*.^27^, ^93^, ^94^, ^95^, ^96^ The *pre‐miR‐423 SNP* can prevent endogenous processing of two mature forms of *miRNA (miR‐423‐5p* and *miR‐423‐3p*).^36^ It is widely believed that miRNA‐SNPs exert biological functions through two main mechanisms: (a) by changing the specific sequence of the miRNA core region and altering the binding affinity between the miRNA and the mRNA; and (b) by affecting the mature modification process of miRNA and altering its expression.^22^

**TABLE 3:** Single nucleotide polymorphisms (SNPs) in premiR‐423 in various tumors

Cancer type	Year	Country	Sample size	Potential significance and sensitivity/specificity	References
Oesophageal squamous cell carcinoma (OSCC)	2013	South Africa	368 cases and 583 controls	In Blacks, under dominant, additive and recessive models with odds ratios (ORs) 1.353, 1.404, and 2.858, respectively. (OR(GE) = 7.855, P(GE) = 9.17*10(−10) in the Black group).	107
	2017	Iran	200cases and 300 controls	Codominant (AA vs. CC, OR [95% confidence interval, CI]: 0.32 [0.15–0.69], *p*‐value: 0.0076); recessive (AA vs. CC+CA, OR [95% CI]: 0.35 [0.16–0.73], *p*‐value: 0.0027); log‐additive models (OR [95% CI]: 0.69 [0.52–0.91], *p*‐value: 0.0084).	109
Esophageal cancer (EC)	2008	USA	346 Case and 346 Control	Per‐allele OR of 0.64 [95% confidence interval: 0.51–0.80; *p* < 0.0001].	106
Esophageal squamous cell carcinoma (ESCC)	2013	China	629 cases and 686 controls	AA vs. CC/CA: adjusted OR = 4.94, 95% CI = 1.42–17.21, *p* = 0.012.	108
	2019	Mixed	22 studies from 13 articles	No significant associations between the rs6505162 and the susceptibility of ESCC in any genetic model.	110
Breast cancer (BC)	2012	Australia	179 cases and 174 controls	The CC genotype reduced risk of breast cancer (OR = 0.50, 95% CI = 0.27–0.92, *p* = 0.03).	100
	2018	United Kingdom	European 97, Asian 90, African 90.	57% Europeans associated with AC genotype, while approx. 60% Asians display mostly associated with CC genotype, and approx. 60% Africans mainly associated with AA genotype.	114
	2015	China.	5 matched cell lines, 114 cases and 114 controls	2/5 Breast cancer cell lines and 8.77% (10/114) tumors undergo somatic mutations of the rs6505162 SNP.	36
	2016	Chile	440 cases and 807 controls	rs6505162:C>A (OR = 1.7 [95% CI 1.0–2.0] *p* = 0.05).	99
	2014	China	7170 case and 8783 controls	rs6505162 no significant association with the risk of breast cancer in any genetic model.	116
	2015	South Korea	136 cases and 224 controls	miR‐423C>A polymorphism is associated with increased risk of POI.	115
Lung cancer (LC)	2016	China	575 case and 608 controls	rs2910164 G allele was associated with lower risk of lung cancer (OR: 0.80), compared with CC genotype (adjusted OR: 0.77) and CA/AA genotype decreased risk (adjusted OR: 0.54). The allele model with OR of 0.75.	102
	2015	China	258 cases and 310 controls	The interaction between rs6505162 gene and environment was not statistically significant.	111
Hepatocellular carcinoma (HCC)	2014	China	984 case and 991 controls	rs6505162 had no effect on the risk of HCC.	119
Advanced gastric cancer (AGC)	2013	Aachen	674 AGC patients	rs6505162 had no effect on the risk of AGC.	121
Wilms tumor (WT)	2018	China	145 cases and 531 controls.	Susceptibility to WT (CA vs. CC: adjusted OR = 0.65, 95% CI = 0.42–0.99, *p* = 0.047). Compared with CC genotype, CA/AA genotypes decreased risk of WT (adjusted OR = 0.30, 95% CI = 0.14–0.63, *p* = 0.002) and clinical stage I+II WT (adjusted OR = 0.42, 95% CI = 0.20–0.85, *p* = 0.017).	28
Colorectal cancer (CRC)	2018	China	117 case and 84 controls	Compared with CC homozygote, the AC heterozygote but not AA homozygote decreased risk of CRC (OR = 0.421, 95% CI = 0.230–0.772, *p* = 0.005 for AC heterozygote; and OR = 0.937, 95% CI = 0.157–5.614, *p* = 0.944 for AA homozygote, respectively), the combination of AC and AA genotype decreased risk of CRC (OR = 0.450, 95% CI = 0.250–0.812, *p* = 0 .008).	118
	2012	China	496 eligible CRC patients	Compared with the homozygous wild‐type genotype, the variant‐containing genotypes of SNP associated with both the OS (HR = 2.12, 95% CI = 1.34–3.34, *p* = 0.001) and the RFS (HR = 1.59, 95% CI = 1.08–2.36, *p* = 0.019).	117
Multiple cancer (meta‐analysis)	2017	Mixed	8582 cases and 10 291 controls	AA genotype decreased cancer risk under the recessive model (OR: 0.87, 95% CI: 0.78–0.98, *p* = 0.020). Subgroup analyses decrease in risk of lung cancer (dominant model: OR = 0.73, 95% CI: 0.60–0.89, *p* = 0.002; recessive model: OR = 0.59, 95% CI: 0.37–0.95, *p* = 0.031).	104
	2018	Mixed	10 500 cases and 13 781 controls	rs6505162 variant significantly decreased the risk of cancer in heterozygous codominant (OR = 0.88, 95% CI = 0.79–0.97, *p* = 0.009, AC vs. CC), dominanat (OR = 0.86, 95% CI = 0.77–0.95, *p* = 0.005, AC+AA vs. CC), and allele (OR = 0.89, 95% CI = 0.82–0.96, *p* = 0.003, A vs. C) genetic models.	92

Abbreviations: 95% CI, 95% confidence interval; OR, odds ratio; OS, overall survival; POI, primary ovarianinsufficiency; RFS, recurrence‐free survival.

Growing evidence supports the association between *rs6505162* and the cancer risk of several cancers, as well as the susceptibility of individuals to cancer, although the results are contradictory.^97^, ^98^, ^99^, ^100^, ^101^, ^102^, ^103^, ^104^ *Pre‐miR‐423 SNP* has been reported to affect mature *miR‐423* expression in cell lines derived from endometrial carcinoma^105^ and breast cancer^36^ tissues, and plays an oncogenic role in tumorigenesis.^36^ The *rs6505162 SNP* has been reported to affect the risk of esophageal cancer.^106^ Many reports have also linked *rs6505162* to environmental exposure to cancer. A recent study conducted in South Africa indicated that *rs6505162* is associated with environmental exposure to smoke and can increase the risk of esophageal squamous cell carcinoma.^107^ In addition to environmental factors, genetic factors (such as *rs6505162*) are also play carcinogenic roles in esophageal squamous cell carcinoma.^108^, ^109^ Nevertheless, a recent study demonstrated that there is no correlation between any genetic model of *miR‐423* (including *rs6505162*) and susceptibility to esophageal squamous cell carcinoma.^110^ Similarly, there is no biological association between *rs6505162* and passive smoking exposure in Chinese women with lung cancer.^111^ The *rs6505162 SNP* is associated with genetic susceptibility to lung cancer and lung adenocarcinoma,^102^ The *rs6505162 SNP* also has implications for the prognosis of patients with completely resected non‐small cell lung cancer.^112^ Two recent meta‐analyses, indicated that *rs6505162* significantly decreased the risk of lung cancer, suggesting that this *miR‐423 polymorphism* is an effective protective factor against lung cancer.^104^, ^113^ Analysis using association methods revealed that the CC genotype of *rs6505162* reduced the risk of breast cancer development.^100^ There are ethnic differences in susceptibility to breast cancer. An in silico analysis showed that the AC genotype is mainly associated with Europeans (57%), while Africans and Asians were mainly AA (about 60%) and CC (about 60%), respectively.^114^ The *rs6505162 SNP* was found to affect the risk of familial breast cancer in a study conducted in South America.^99^ Rah et al reported that breast cancer‐related *microRNA* polymorphisms, including *rs6505162*, are connected with increased primary ovarian insufficiency risk via interactions with *miR‐423* A variants.^115^ Interestingly, a case‐control study (7170 breast cancer and 8783 controls) showed no correlation between the various genetic models of *miR‐423* and the risk of breast cancer.^116^ Dozens of colorectal cancer susceptibility bases have been identified by genome‐wide association analysis (GWAS), and *rs6505162* has been shown to affect the prognosis of colorectal cancer patients following chemotherapy.^63^, ^117^ Similarly, *rs6505162* has been identified as a biomarker of colorectal cancer metastasis.^118^ Another study indicated that *rs6505162* has a negative impact on susceptibility to Wilms tumor, especially in Chinese children.^28^ However, no significant associations were found in some cancers, such as bladder cancer,^103^ HCC,^119^ and gastric cancer/advanced gastric cancer.^120^, ^121^ The *miR‐423 rs6505162* polymorphism is considered to be a tumor‐related susceptibility gene. Functional characterization of the *rs6505162* polymorphism as potentially harmful risk alleles helps clarify its function in complex diseases.

## 5 CLINICAL IMPLICATIONS OF *MiR‐423* IN CANCER

MiRNA plays an irreplaceable regulatory role in the occurrence and development of tumors.^122^ Specific serum miRNA expression profiles can be used for early diagnosis of cancer as well as monitoring treatment efficacy.^123^ High *miR‐423* expression in glioma patients may indicate advanced grade and poor prognosis.^46^, ^67^ The combination of *miR‐423‐5p* with other *miRNA* biomarkers (*miR‐150‐5p and miR‐222‐3p*) can be used to monitor the malignant progression of oral leukoplakia.^124^, ^125^ Overexpression of *miR‐423‐5p* has been implicated as a biomarker or therapeutic target of brain metastasis in lung cancer.^60^ The combination of *miR‐423‐3p* and the three other *miRNAs* (*miR‐148b*, *miR‐221* and *miR‐23b*) has proved to be a novel and non‐invasive marker for lung cancer detection, with an area under curve (AUC) of 0.885.^126^ Therefore, *miR‐423* is expected to become a therapeutic target and prognostic marker for lung cancer.^58^ *MiR‐423* plays a similar prognostic role in HCC patients. When *miR‐423* was used in combination with three other miRNAs (*miR‐10a*, *miR‐375*, and *miR‐122*), the AUC for the diagnosis of HCC was 0.995.^127^ The combination of *miR‐423‐3p* with *miR‐151‐5p* and *miR‐152‐3p* is also a clinically effective marker for cervical cancer patients.^128^ In patients with stage I–II colorectal cancer, serum *miR‐423‐5p* levels were significantly upregulated compared with the levels in healthy controls, whereas in stage III–IV patients, there was no significant increase in serum *miR‐423‐5p* levels.^129^ Therefore, plasma levels of *miR‐423‐5p* can be used as a biomarker for early detection of colorectal cancer.^49^, ^130^ *MiR‐423‐5p* also has a similar function and clinical prognostic value in ovarian cancer.^38^ The miRNAs in different ethnic breast cancers, as well as specific genetic variations in miRNA genes, may influence the risk of breast cancer.^114^ It has been reported that 70.1% of hereditary breast cancers can be distinguished from non‐hereditary breast cancer on the basis of *miR‐423‐3p* expression.^131^ Identification of the 7‐miRNA group (including *miR‐423‐5p*) has improved the accuracy of diagnosing bladder cancer with an AUC of 0.923, of which urinary miRNAs may have important clinical value in the prediction of recurrence in bladder cancer.^132^ *MiR‐423‐3p* can be used in combination with four other *miRNAs* (*miR‐1*, *miR‐20a*, *miR‐27a*, and *miR‐34*) to diagnose gastric cancer, with the AUC reaching 0.879, which is significantly higher than that of *CA19‐9* (0.600) and *CEA* (0.503).^133^ Similarly, serum exosome‐derived *miR‐423* may also be a new target for gastric cancer‐specific therapeutic interventions.^31^, ^34^, ^52^ In summary, clinical studies of *miR‐423* in human cancer are still in the preliminary stages, and further large‐scale evidence‐based medical research is needed to confirm the clinical importance of *miR‐423*.

## 6 CONCLUSION

In this review, we have introduced published literature on the dysregulation of *miR‐423* in malignancies and the molecular mechanism of *miR‐423* mediated carcinogenesis. Expression of *miR‐423* has been confirmed to be abnormal in various human cancers and has been revealed as a critical factor involved in tumorigenesis. Although clinical applications of *miR‐423* in cancer are yet to be established, accumulating evidence supports the use of *miR‐423* as a biomarker in the early diagnosis and prognosis of human cancers in addition to monitoring treatment efficacy.

## COMPETITIVE INTERESTS

The authors claim that they have no competing interests.
